# Layered Perovskite Doping with Eu^3+^ and β-diketonate Eu^3+^ Complex

**DOI:** 10.1021/acs.chemmater.0c04097

**Published:** 2021-03-21

**Authors:** Daniele Cortecchia, Wojciech Mróz, Giulia Folpini, Tetiana Borzda, Luca Leoncino, Ada Lili Alvarado-Leaños, Emily Mae Speller, Annamaria Petrozza

**Affiliations:** † Centre for Nano Science and Technology (CNST@PoliMi), Istituto Italiano di Tecnologia, Milan 20133, Italy; ‡ Electron Microscopy Facility, Istituto Italiano di Tecnologia, Via Morego 30, Genova 16163, Italy

## Abstract

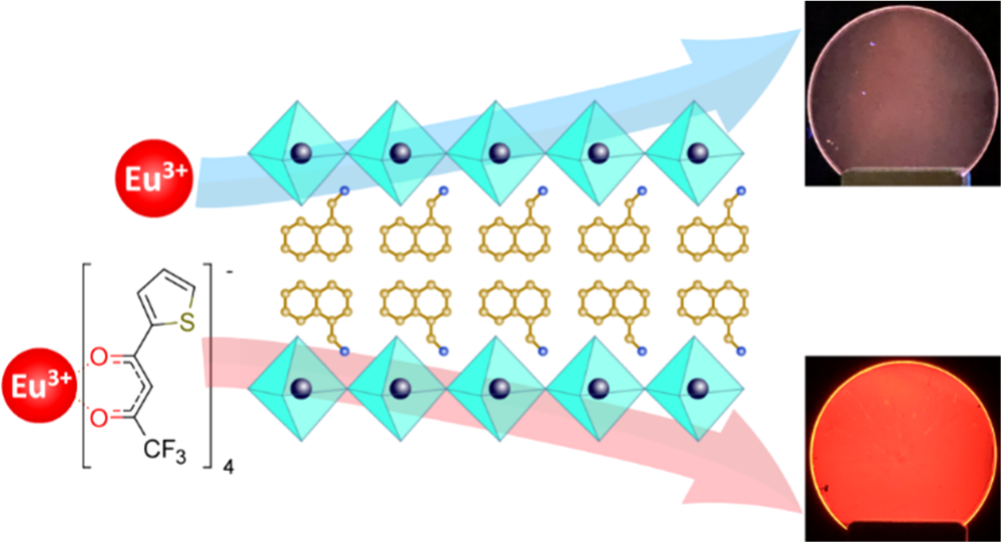

Metal halide perovskites are attracting great interest for the fabrication of light-emitting devices encompassing light-emitting diodes, lasers, and scintillators. As the field develops, perovskite doping emerges as a promising way to enrich the material functionalities and enhance the luminescence yield and tunability. While Mn^+2^ addition has been well explored, doping with lanthanides has received less attention, even though their intense and line-like luminescence is interesting for a wide range of applications. In this work, we study the doping of NMA_2_PbBr_4_ layered perovskites with Eu^3+^ and Eu^3+^ tetrakis β-diketonate complex. By exploiting the antenna effect of the naphthalene-based functional cation (NMA = 1-naphtylmethylammonium), direct sensitization of Eu^3+^ is obtained; nevertheless, it is not very efficient due to the non-optimal energy level alignment with the resonance acceptor level of the lanthanide. Protection of Eu^3+^ in the form of tetrakis β-diketonate complex grants a more ideal coordination geometry and energetic landscape for the energy transfer to europium in the perovskite matrix, allowing for a nearly 30-fold improvement in luminescence yield. This work sets the basis for new synthetic strategies for the design of functional perovskite/lanthanide host–guest systems with improved luminescence properties.

## Introduction

Metal halide perovskites (MHPs) are attracting great interest for a wide range of light-emitting applications including light-emitting diodes (LEDs) and transistors,^1,2^ lasers and scintillators.^3,4^ The surge in device performance strongly motivates the investigation of synthetic strategies to further enrich the properties of MHPs and provide them with new functionalities.^5^ Considering the three-dimensional (3D) MHP structure with general formula ABX_3_, B-site doping with d-block and f-block elements has been pursued to boost the luminescence properties. CsPbCl_3_ nanocrystals has been the most investigated host, although in a few cases successful doping have been reported also in layered perovskites.^5−9^ Among the notable examples, energy transfer to Mn^2+^ leads to broadband orange luminescence with quantum yield up to 30–40% and has been employed for LEDs and luminescent solar concentrators.^8,10,11^ Within lanthanides, Yb^3+^, Yb^3+^/Er^3+^, and Yb^3+^/Ce^3+^ have been the most studied systems since they allow efficient NIR luminescence with extraordinary photoluminescence quantum yields (PLQYs) surpassing 100% aided by a quantum cutting process.^12−15^ Doping with other rare-earth metals, despite a few promising attempts,^8,14^ has not been thoroughly investigated. Among these, trivalent europium (Eu^3+^) has a characteristic intense red luminescence which makes it very interesting for light-emitting devices with high color purity,^16,17^ optical amplifiers and lasers,^18,19^ sensors and spectroscopic probes.^20^ Since the closed 5s^2^ and 5p^6^ shells efficiently screen the partially filled 4f shell, where the transitions responsible for the luminescence occur, the electronic configuration is only weakly perturbed thus resulting in extremely narrow emission lines and long excited state lifetimes.^21,22^ Eu^3+^ doping has been shown in combination with a wide range of host matrices like glasses, xerogels, polysilsesquioxanes, metal organic frameworks, polyoxometalates, organic polymers as well as in intercalation compounds with layered materials, such as layered double hydroxides and clays.^23^ Concerning MHPs, europium has been used to improve the operational and phase stability of perovskite solar cells^24,25^ and employed to expand the luminescence properties of CsPbCl_3_ and CsPbBr_3_ nanocrystals for application in LEDs.^14,26−30^ However, europium doping in low-dimensional MHPs and the use of functional organic cations to improve the lanthanide sensitization within the perovskite framework have been scarcely investigated.

Compared to 3D perovskites, layered perovskites offer greater synthetic and structural flexibility which are beneficial to carefully explore the photophysical and structural design required to achieve efficient luminescence through Eu^3+^ doping.^31^ In lanthanides, intraconfigurational electric dipole (ED) f–f transitions are strictly spin and parity forbidden. Selection rules are partially relaxed by spin–orbit coupling and crystal-field perturbations which induce mixing of the levels of the 4f configuration with orbitals having opposite-parity wavefunctions, such as 5d orbitals.^21,22,32^ Even though these mechanisms allow ED transitions to occur, Eu^3+^ (likewise other lanthanide ions) suffers from very low absorption coefficients, in turn resulting in weak luminescence. This issue is typically solved by (1) including Eu^3+^ in a strongly asymmetric site of the host matrix^33^ and (2) sensitizing Eu^3+^ with organic ligands, where their larger absorption coefficient is exploited to more efficiently absorb light and transfer energy to the lanthanide (antenna effect),^34^ with the energy transfer process typically involving a nonradiative transition from the triplet state of the organic ligand to an excited state of Eu^3+^.^21^ To populate a resonance level of Eu^3+^, there should be an overlap between the ligand phosphorescence and the absorption of the lanthanide, and the triplet of the ligand should be positioned at an energy above a resonance level of Eu^3+^.^20^ Second, the lanthanide ion should be protected from non-radiative deactivation of excited states through vibration of the lattice or ligands.^21^ An in-depth understanding of the transfer mechanisms in Eu^3+^ excitation is a crucial step for the achievement of high luminescence efficiencies.

Here, we study two-dimensional (2D) perovskites doping by means of Eu^3+^ ions and a europium chelate. We show that functional organic molecules can be exploited to provide effective sensitization of the lanthanide ion embedded in the perovskite matrix. Naphthalene-based derivatives, such as naphthoyltrifluoroacetone and naphthalene-functionalized cyclen, have been used in the past as antennae to populate the Eu^3+^ excited state.^35−37^ In this case, we directly employ NMA = 1-naphtylmethylammonium as a templating cation to form the perovskite host NMA_2_PbX_4_ (where X = Br,Cl) and discuss its role in the sensitization of Eu^3+^ by means of temperature and power-dependent measurements and time-resolved photoluminescence (TRPL) spectroscopy. Although lanthanide luminescence is observed with direct B-site doping, better population of Eu^3+^ excited states is achieved by protection of Eu^3+^ in the form of tetrakis β-diketonate complex,^17^ which leads to the formation of an intercalation compound in the layered structure of the perovskite with nearly 30-fold improvement in luminescence quantum yield. This work expands the range of synthetic tools for the development of functional perovskites with improved luminescence properties, with great relevance for applications in light-emitting and photonic devices.

## Results and Discussion

**Figure 1:**
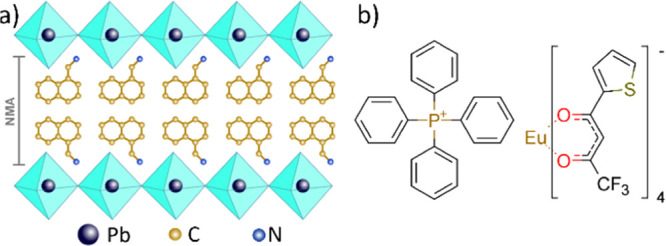
(a) Schematic representation of NMA_2_PbBr_4_ and (b) europium complex Eu(tta)_4_P(Ph)_4_.

NMA_2_PbBr_4_ (Figure 1a) films were synthetized by spin-coating of dimethylformamide (DMF) solutions of stoichiometric amounts of the precursors (NMA)Br and PbBr_2_.

Two doping strategies were pursued: (1) substitutional doping with Eu^3+^ ions to form NMA_2_PbBr_4_:Eu was achieved by addition of EuCl_3_ to the perovskite spin-coating solution; (2) doping with europium chelate was obtained by initially synthetizing the tetrakis β-diketonate complex Eu(tta)_4_P(Ph)_4_ [hereinafter Eu(L); Figure 1b], where Eu^3+^ is coordinated by the four ligands tta = thenoyltrifluoroacetonate, thus resulting in an anionic complex, with the electric neutrality maintained by the tetraphenylphosphonium P(Ph)_4_ counter ion. Note that the ligand choice has been also driven by the following key requirements: (1) compatibility with the perovskite processing conditions, including good thermal stability to sustain the annealing process; (2) compatibility with the ionic perovskite host; and (3) spectral match with the perovskite’s luminescence (a necessary condition to realize energy transfer from the perovskite to the complex). Eu(tta)_4_P(Ph)_4_ satisfies all these conditions. The ionic nature of the tetrakis complex favors its retention in the perovskite ionic lattice by coulombic interaction. The preformed complex is added to the spin-coating solution to form the doped system NMA_2_PbBr_4_:Eu(L). In both cases, doping was performed by adding a 10% mol content of Eu^3+^ with respect to Pb^2+^.

**Figure 2:**
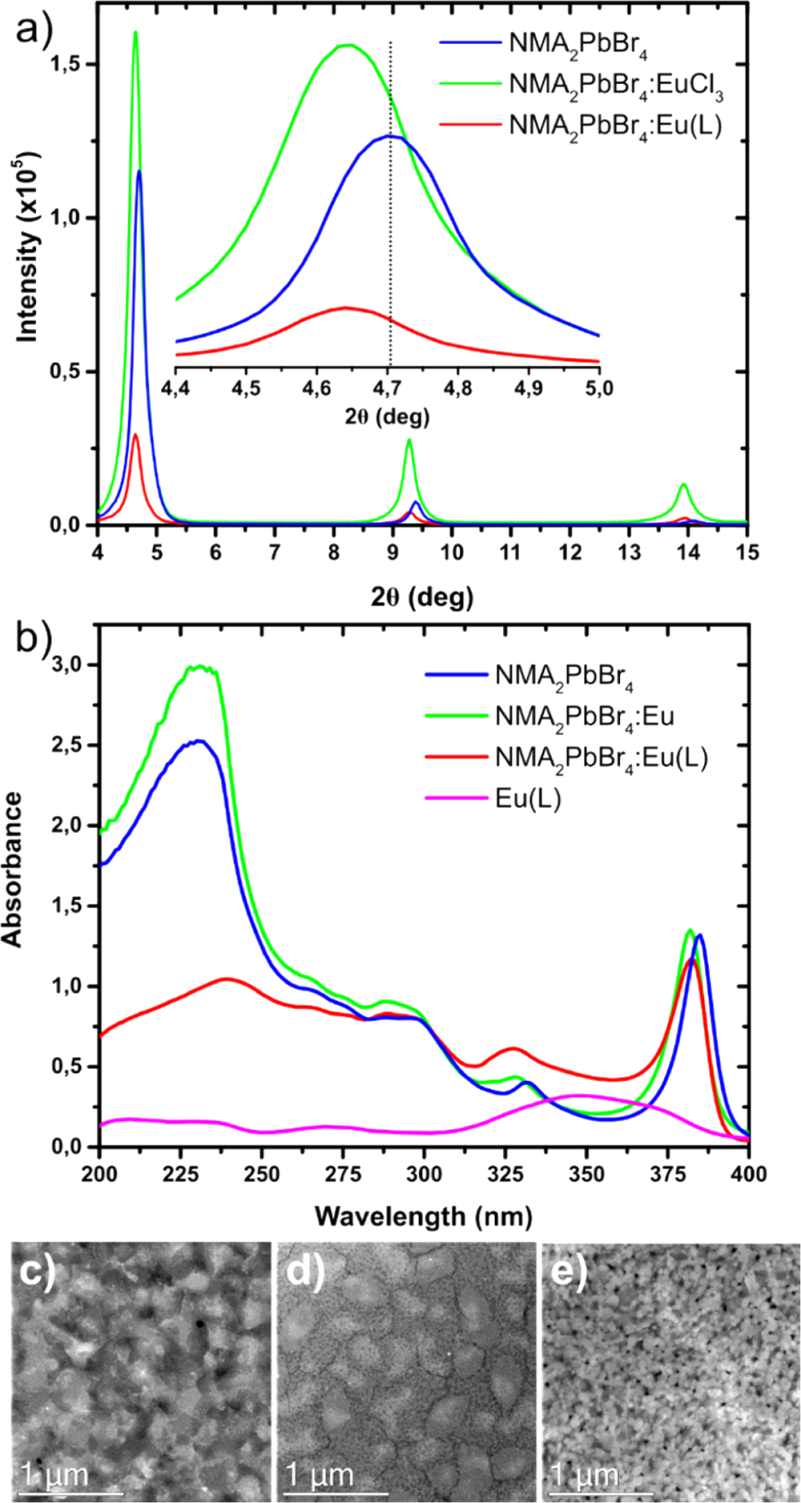
(a) XRD pattern and (b) absorption spectra of perovskite thin films. High-angle annular dark-field STEM (HAADF-STEM) images of (c) NMA_2_PbBr_4_, (d) NMA_2_PbBr_4_:Eu, (e) NMA_2_PbBr_4_:Eu(L).

Figure 2a shows the X-ray diffraction (XRD) patterns of the obtained thin films. Due to the strong preferential orientation typical of 2D perovskites, only three diffraction peaks (4.7, 9.38, and 14.1°) are observed for NMA_2_PbBr_4_. The retention of the same diffraction pattern in NMA_2_PbBr_4_:Eu and NMA_2_PbBr_4_:Eu(L) indicates that the perovskite is formed also in the presence of the dopants. The weaker diffraction of NMA_2_PbBr_4_:Eu(L) suggests a reduction in crystallinity, as can be expected by the introduction of the bulky complex. In both cases, we also observe a small but clear shift of the diffraction peaks to smaller angles (inset in Figure 2a), indicating the increase in interplanar distance. While this trend can be explained by partial incorporation of chlorine in NMA_2_PbBr_4_:Eu due to the addition of EuCl_3_ (see Figure S1), lattice expansion is particularly interesting for NMA_2_PbBr_4_:Eu(L), where it points to the formation of an intercalation compound similar to other perovskites incorporating organic luminophores.^38^ From XRD, we estimate an interplanar distance between the adjacent inorganic layers of 18.8 Å in NMA_2_PbBr_4_, which is comparable to that reported for (2-NMA)_2_PbBr_4_, where 2-NMA = 2-naphtylmethylammonium.^39^ Considering a diameter of about 11 Å for [Eu(tta)_4_]^−^,^40^ it is possible for the europium complex to intercalate within the perovskite layers by inducing disorder of the organic cations as well as local widening of the interplanar distance. Absorption spectra (Figure 2b) further support this assumption. In all of the three compounds, only minimal differences are found in the excitonic absorption peak around 385 nm, indicating that the inorganic framework is largely preserved. On the contrary, a big reduction in absorbance is found for NMA_2_PbBr_4_:Eu(L) below 275 nm, a region dominated by the π–π* transitions of the organic cation NMA (Figure S2), which can be attributed to a strong disruption of the NMA stacking pattern and modifications in the intermolecular forces holding the crystal upon insertion of Eu(L), which are likely to affect the nature of the collective excitations of the resulting solid.^41−43^ Incorporation of Eu(L) is evident by the increase in absorbance around 350 nm in NMA_2_PbBr_4_:Eu(L) and confirmed by Fourier-transform infrared (FTIR) spectra (Figure S3).

We further characterized the materials using transmission electron microscopy (TEM) operating in scanning TEM (STEM) mode combined with energy dispersive X-ray spectroscopy (EDS) (Figures 2c–e and S4–S8). Compared to the other two systems, NMA_2_PbBr_4_:Eu(L) exhibits smaller grains which likely helps to release the lattice strain induced by the inclusion of the bulky complex. Eu/Pb molar ratios of around 12 and 14% were found in NMA_2_PbBr_4_:Eu and NMA_2_PbBr_4_:Eu(L), respectively, in good agreement with the starting stoichiometry (Figures S5 and S6). Fluorine and phosphorus were also detected in NMA_2_PbBr_4_:Eu(L), which was indicative of the presence of the europium complex ligand and counter ion (Figure S6). In all three samples, we found non-uniformities at the sub μ-length scale which are attributed to Pb-rich regions, likely related to the precipitation of Pb nanoparticles (Figures S7 and S8). Apart from these, both the STEM images and the elemental maps did not reveal significant phase segregations, indicating a uniform distribution of the dopant across the sample, good matrix/dopant intermixing, and absence of dopant clustering at the grain boundaries.

**Figure 3:**
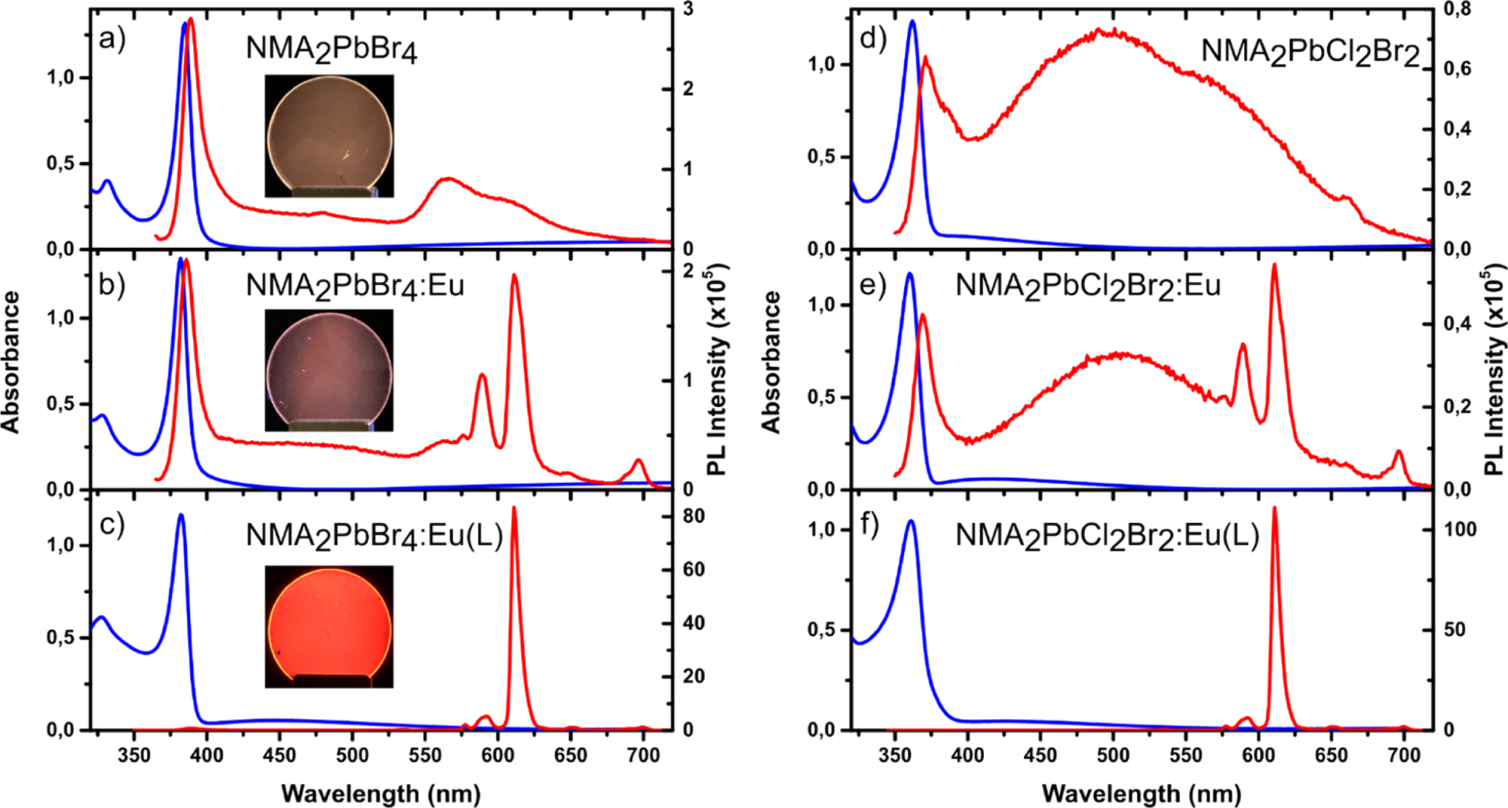
Absorption (blue) and PL (red) spectra of pristine and doped perovskites. For PL measurements, pump excitations of 350 and 330 nm are used for the series of NMA_2_PbBr_4_ and NMA_2_PbCl_2_Br_2_ perovskites, respectively. The insets show the luminescence of perovskite films under 350 nm excitation.

Figure 3 compares the steady-state PL employing 10% mol content of Eu^3+^; the trends for different doping concentrations are shown in Figure S9. NMA_2_PbBr_4_ is characterized by the narrowband excitonic emission at 389 nm and a secondary band peaked at 564 nm related to the NMA triplet emission.^44^ After introduction of Eu^3+^ ions, the five sharp emission bands peaking at 576, 589, 611, 648, and 697 nm, characteristic of the Eu^3+^ intraconfigurational transitions ^5^D_0_ → ^7^F_*J*_ (*J* = 0–4) appear overlapped with the triplet emission. Among these, *J* = 0,2,3,4 are forced ED transitions, implying that they are strictly forbidden by selection rules for purely electronic transitions when Eu^3+^ occupies a site with strict center of symmetry. On the contrary, ^5^D_0_ → ^7^F_1_ is a magnetic dipole transition, which is forbidden only by spin but allowed by the Laporte selection rule.^33^ As such, in a strictly octahedral crystal field, only the ^5^D_0_ → ^7^F_1_ transition at 589 nm is expected to be observed.^20^ Doping with Eu^3+^ proceeds with partial substitution of Pb^2+^ so that Eu^3+^ would occupy an octahedral site. The fact that all five transitions are visible in the PL spectrum is evidence that strong deviations from the inversion symmetry is reached,^33^ which can be derived from concomitant contributions involving strong octahedral distortion, the spatial arrangement of the nearest neighbors (e.g., organic cations), and the symmetry group of the crystal lattice. In fact, the appearance of the weak ^5^D_0_ → ^7^F_0_ at 576 nm is further indication that Eu^3+^ occupies a site with either C_nv_, C_n_, or C_s_ symmetry.^20^ We also explored the tuning of the host band gap by partial substitution of Br with Cl using the stoichiometry NMA_2_PbCl_2_Br_2_ (Figure 3d,e). Chlorine addition causes a 20 nm blueshift of the excitonic absorption and PL, jointly with the appearance of a very broad band peaked at 500 nm characteristic of trap emission in layered perovskites;^31^ here, NMA triplet emission is observed as a shoulder at 560 nm. Despite the band gap shift, emission from Eu^3+^ is not significantly changed (Figure 3e), suggesting that the host band gap does not impact the transfer efficiency, contrary to what was previously observed with Mn^2+^.^8^ In order to understand the possible role of the NMA triplet in the energy transfer, we synthetized europium-doped BA_2_PbCl_*x*_Br_4–*x*_ and PEA_2_PbCl_*x*_Br_4–*x*_ (BA = butylammonium and PEA = phenethylammonium), where the organic cation does not have an accessible triplet level. In all these systems, Eu^3+^ luminescence is not observed (Figures S10–S12), indicating that the antenna effect from NMA is crucial to sensitize the lanthanide. In fact, we do observe partial quenching of the NMA phosphorescence upon europium doping (Figures 3 and S9 and S10). However, the clear presence of residual emission from NMA indicates that the transfer is not complete, thus limiting the PLQY which remains low (about 0.3%, comparable to the undoped material). To provide a more optimized energy level alignment for the transfer to europium, as well as a rigid metal-ion environment preventing de-excitation of the excited state through coupling with lattice vibrations, we protected Eu^3+^ with tta ligands. Once the complex is formed, the ^5^D_0_ → ^7^F_2_ transition at 611 nm is strongly enhanced and dominates the bright red emission of NMA_2_PbBr_4_:Eu(L) and NMA_2_PbCl_2_Br_2_:Eu(L) (Figure 3c,f). ^5^D_0_ → ^7^F_2_ is called the “hypersensitive transition”^20^ as it is very sensitive to the nature of the ligands surrounding Eu^3+^ and its local geometry. The intensity ratio *R* = *I*(^5^*D*_0_ → ^7^F_2_)/*I*(^5^D_0_ → ^7^F_1_) is 1.8 and 16.2 for NMA_2_PbBr_4_:Eu and NMA_2_PbBr_4_:Eu(L), respectively, as a result of the strong asymmetry imparted by tta ligands and their higher polarizability compared to the halides surrounding Eu^3+^ in NMA_2_PbBr_4_:Eu. The narrower emission of NMA_2_PbBr_4_:Eu(L) also suggests a weaker coupling with the vibrations of the host matrix. Emission from the perovskite can be exploited to excite the complex if spectral overlap between the perovskite emission and Eu(L) absorption is realized.^16^ This occurs only partially in NMA_2_PbBr_4_, while a better spectral overlap can be achieved by chlorine addition (Figure S13); however, the emergence of the broadband Stokes-shifted emission at high chlorine contents limits the extent to which this strategy can be used. We obtained PLQYs of 9 and 13% for NMA_2_PbBr_4_:Eu(L) and NMA_2_PbCl_2_Br_2_:Eu(L), respectively, which represent a ∼30-fold enhancement compared to that of NMA_2_PbBr_4_:Eu.

**Figure 4:**
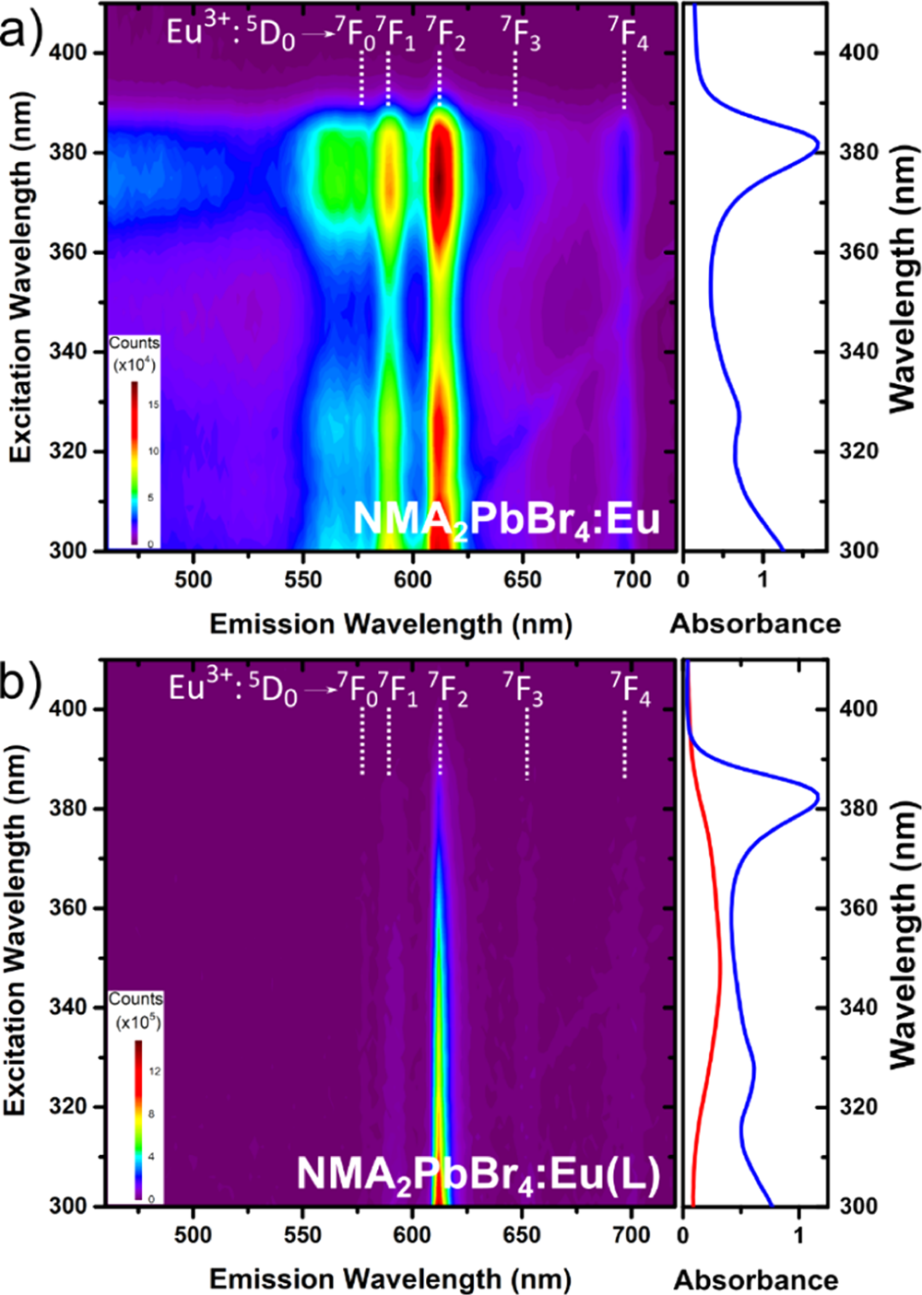
Photoexcitation maps of (a) NMA_2_PbBr_4_:Eu and (b) NMA_2_PbBr_4_:Eu(L). The blue lines show the absorption spectra of the corresponding perovskite, while the red line shows the absorption of the Eu(L) complex.

The photoexcitation map of NMA_2_PbBr_4_:Eu shows an excellent match of the perovskite absorption profile with both the NMA triplet emission and europium luminescence (Figure 4a; the photoexcitation map of the undoped NMA_2_PbBr_4_ is shown in Figure S14). This is in agreement with a process involving energy funnelling from the excitons in the [PbBr_4_]^2–^ layers to the organic sheets and final transfer from the NMA triplet to the lanthanide. On the other hand, NMA_2_PbBr_4_:Eu(L) shows a different photoexcitation profile (Figure 4b), with the europium emission tracing the absorption profile of the Eu(L) complex, indicating that Eu sensitization is mainly derived from the tta ligands directly chelating the metal ion.

**Figure 5:**
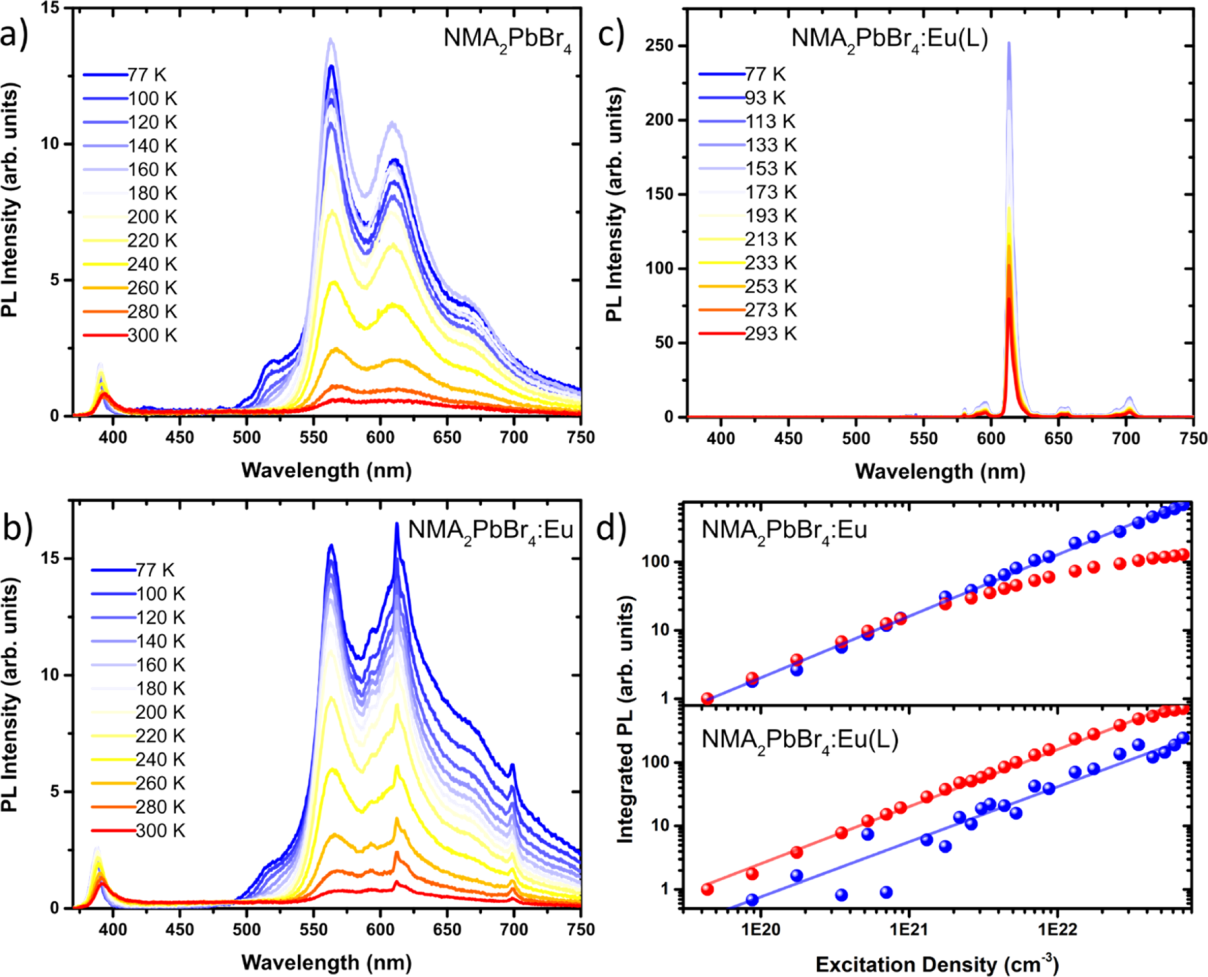
Temperature-dependent PL of (a) NMA_2_PbBr_4_, (b) NMA_2_PbBr_4_:Eu, and (c) NMA_2_PbBr_4_:Eu(L). (d) Integrated PL intensity, at 77 K, as a function of excitation density for NMA_2_PbBr_4_:Eu (upper panel) and NMA_2_PbBr_4_:Eu(L) (lower panel). The data shown in blue are related to the excitonic emission (integration in the range 380–480 nm), while the data in red color show the integration in the range 480–900 nm (see Figure S15 for the corresponding spectra). Curves in 5d have been shifted on the *y*-axis to allow a better comparison. Solid lines show the linear fitting to the experimental data with the power law dependence *I* = *P*^*K*^; for NMA_2_PbBr_4_:Eu *K* = 0.90 ± 0.01 (blue line), while for NMA_2_PbBr_4_:Eu(L) *K* = 0.90 ± 0.01 (red line), and *K* = 0.87 ± 0.05 (blue line).

**Figure 6:**
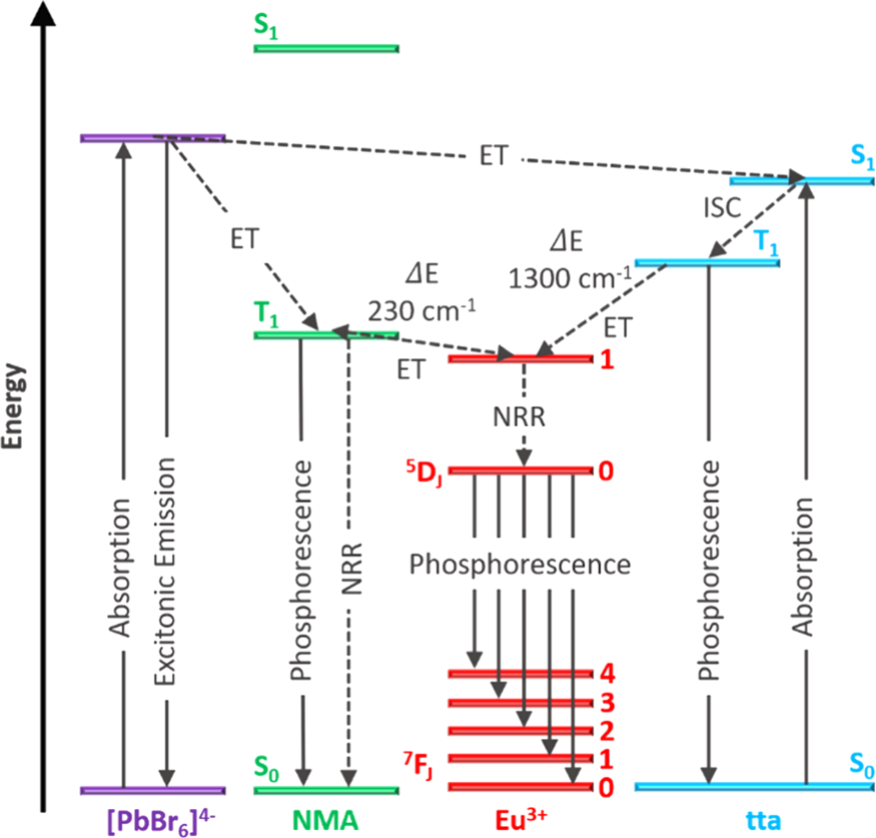
Schematic energy level diagram showing the main transitions occurring in the doped systems. Trapping processes and trap emission from the perovskite are not represented. The energy levels of tta are from external refs (19) and (37). S = singlet state; T = triplet state; ET = energy transfer; NRR = non-radiative relaxation; ISC = intersystem crossing.

To clarify the transfer processes involved in these systems, we performed temperature- and power-dependent PL measurements (Figure 5). At low temperatures, the NMA triplet emission in NMA_2_PbBr_4_ gains strength against the excitonic emission, which is useful to identify the emission onset (Figure 5a): we estimate the NMA triplet energy from the shortest-wavelength phosphorescent band centered around 520 nm (19,230 cm^–1^).^37,45−47^ The best resonance acceptor level of Eu^3+^ is ^5^D_1_, located about 19,000 cm^–1^ (526 nm) above the ^7^F_0_ ground state, from which radiationless decay to the ^5^D_0_ emitting level occurs before undergoing radiative recombination.^20,37^ The main processes and feeding levels involved in the Eu^3+^ sensitization and the Eu^3+^ f–f radiative transitions are summarized in Figure 6. Although the NMA triplet (*T*_NMA_) in NMA_2_PbBr_4_ has the right energetics to populate the ^5^D_1_ level of europium, the very small energy gap *T*_NMA_ – ^5^D_1_ = 230 cm^–1^ makes back transfer very favorable, thus causing strong residual phosphorescence.^37,48,49^ In fact, it is experimentally demonstrated that the triplet level of the sensitizer should be located around 1500 cm^–1^ above the acceptor level to achieve efficient transfer.^20,37^ In comparison, the triplet level of tta (*T*_tta_) is located at 20,300 cm^–1^ (492 nm),^19,37^ and the energy gap *T*_tta_ – ^5^D_1_ = 1300 cm^–1^ is therefore more favorable for Eu^3+^ sensitization (Figure 6). Low-temperature PL of NMA_2_PbBr_4_:Eu (Figure 5b) shows that both the NMA phosphorescence and Eu^3+^ luminescence remain strongly competing processes down to 77 K, thanks to the thermal deactivation of the nonradiative decay pathways affecting the organic cation.^48,50^ In stark contrast, the europium luminescence dominates the emission spectrum of NMA_2_PbBr_4_:Eu(L) throughout the entire temperature range (Figure 5c), confirming the efficient sensitization imparted by the β-diketonate ligand. Previous works on lanthanide chelates have shown that the energy transfer takes place through an electron exchange mechanism, which is strongly dependent on the donor–acceptor distance.^20^ In the more efficient NMA_2_PbBr_4_:Eu(L), the β-diketonate ligands help to minimize the distance by directly chelating the metal ion, with a Eu–O bond length in the order of 2.4 Å.^40^ On the other hand, in NMA_2_PbBr_4_:Eu, we expect NMA to be in the second sphere of coordination of Eu^3+^, with an estimated distance of 4.5 Å between Eu^3+^ and the NMA ammonium moiety,^39^ thus representing an additional barrier to an efficient transfer. To better capture the transfer dynamics, we also performed excitation density-dependent PL measurements at 77 K (see the spectral evolution in Figure S15); the corresponding integrated spectra are shown in Figure 5d in double logarithmic scale. The excitonic emission in NMA_2_PbBr_4_:Eu follows the power law dependence *I* = *P*^*K*^ (where *I* and *P* indicate the PL intensity and excitation power, respectively) with *K* ≲ 1, as expected for excitonic emission with a contribution from non-radiative decay paths, such as transfer to the NMA triplet state or recombination on defects. On the other hand, the PL integrated in the range 480–900 nm (red curve in Figure 5d) clearly deviates from the linear behavior and tends to flatten at high excitation densities; the same is observed also when narrower ranges are considered, isolating the emission from either the NMA triplet or Eu^3+^ (see Figure S16). This trend suggests the saturation of NMA triplet states, which likely act as a bottleneck for the effective europium sensitization. In NMA_2_PbBr_4_:Eu(L), at high excitation densities, the weak excitonic emission becomes more easily visible and we also note the presence of two additional europium-based transitions (Figure S15), ^5^D_0_ → ^7^F_5_ (750 nm) and ^5^D_0_ → ^7^F_6_ (820 nm), which are rarely reported due to their weakness in molecular compounds. Here, the europium emission shows a linear power dependence similar to that of the excitonic emission (Figure 5d), which is indicative of an efficient transfer to the lanthanide ions.

**Figure 7:**
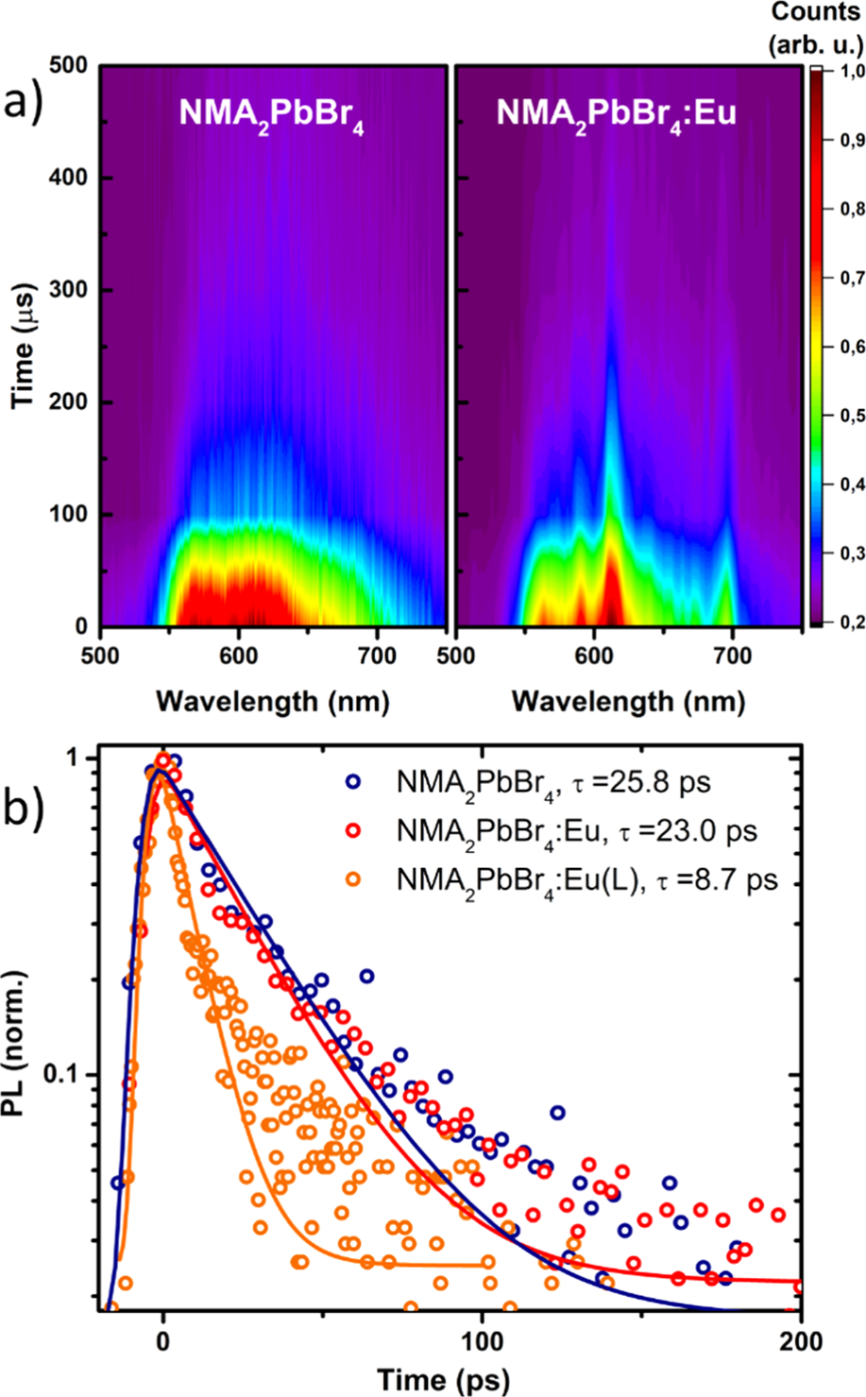
(a) Time-resolved PL maps of the NMA phosphorescence and europium luminescence in NMA_2_PbBr_4_ (left panel) and NMA_2_PbBr_4_:Eu (right panel). (b) Comparison of the exciton decay dynamics in the range 380–395 nm for the doped and undoped NMA_2_PbBr_4_ perovskite. The solid lines show the result of a monoexponential decay fit.

Time-resolved PL shows that both the NMA and Eu emissions are characterized by slow decay dynamics with lifetimes extending to few hundred μs, with the lanthanide being slightly longer-lived (Figure 7a). This timescale is characteristic for molecular phosphorescence and for the forbidden nature of the Eu^3+^ f–f intraconfigurational transitions. Due to the strong residual phosphorescence and the limit of our temporal resolution, we could not probe a clear NMA quenching even after europium addition. The slow decay of the NMA phosphorescence, much slower than the perovskite exciton dynamics falling in the ps range (Figure 7b), corroborates the possible saturation of NMA triplets suggested by power-dependent measurements. The excitonic emission has comparable lifetimes (around 25 ps, monoexponential fit results are presented in Supporting Information, Table S1) in both NMA_2_PbBr_4_ and NMA_2_PbBr_4_:Eu, indicating that the exciton decay dynamics are dictated by the transfer to the NMA cations and not further affected by the addition of the lanthanide. On the contrary, a more effective quenching is found in NMA_2_PbBr_4_:Eu(L), with the exciton lifetime decreasing to 8 ps upon addition of the complex, likely a consequence of partial energy transfer from the perovskite to the Eu(L) complex (Figure 6).

We finally note that the sharp hypersensitive 5D_0_ → ^7^F_2_ transition dominates also the electroluminescence spectrum of NMA_2_PbBr_4_:Eu(L), proving that the lanthanide complex embedded in the perovskite matrix can also be excited electrically (Figure S17). This highlights the possibility to exploit doping with lanthanide complexes as a strategy to improve the tunability and color purity of perovskite light-emitting devices. However, further research is necessary to improve the out-of-plane charge transport and charge injection in the wide band gap perovskites, which typically limit the efficiencies of single-layered 2D perovskite-based devices.^8^

## Conclusions

Here, we have investigated doping of 2D perovskites with europium, either by direct substitutional doping with Eu^3+^ ions or by protection of Eu^3+^ in the form of the tetrakis β-diketonate complex Eu(tta)_4_P(Ph)_4_, Eu(L). In NMA_2_PbBr_4_:Eu, sensitization of the lanthanide is achieved by exploiting the antenna effect from the functional cation NMA = 1-naphtylmethylammonium. However, the non-ideal energy level alignment and binding geometry prevents the complete energy transfer to Eu^3+^ and hampers the luminescence efficiency (PLQY = 0.3%). Protection of Eu^3+^ in the form of a complex Eu(L) allows us to optimize the energetic landscape for Eu^3+^ sensitization with consequent improvement in luminescence yield, with PLQY reaching 9% in NMA_2_PbBr_4_:Eu(L). This work expands the range of synthetic strategies for designing functional MHPs and sets the basis for further developments. We expect that higher luminescence yields will be achieved either through the improvement of the energy transfer from the perovskite host to the europium complex by optimizing the resonance conditions of the donor/acceptor system or by adopting perovskite templating cations which can more efficiently populate the lanthanide excited state. In this regard, the formation of lanthanide-based paddle-wheel clusters linking perovskite layers represents a promising avenue.^51^ Furthermore, the possibility to electrically excite lanthanide complexes in the perovskite matrix opens the way to their application in perovskite light-emitting devices with improved color purity.

## Experimental Section

### Materials

1-Naphtylmethylamine (97%, Sigma-Aldrich), lead bromide PbBr_2_ (TCI), lead chloride PbCl_2_ (99.999% Sigma-Aldrich), europium(III) chloride EuCl_3_ (99.99% Sigma-Aldrich), EuCl_3_·6H_2_O (99.9% Sigma-Aldrich), DMF (anhydrous, Sigma-Aldrich), hydrobromic acid HBr (48% in water, Sigma-Aldrich), 4,4,4,-trifluoro-1-(2-thienyl)-1,3-butadione (Htta, thenoyltrifluoroacetone) (99%, Sigma-Aldrich), tetraphenylphosphonium chloride P(Ph)_4_Cl (98%, Sigma-Aldrich), sodium hydroxide NaOH (Sigma-Aldrich), tetrahydrofuran THF (99.9%, Sigma-Aldrich), and dichloromethane DCM (anhydrous, Sigma-Aldrich).

### Synthesis of 1-NMA Bromide (NMA)Br

1-Naphtylmethylamine (1.5 mL, 0.01 mmol) was dissolved in 40 mL of THF, and 3.3 mL of HBr 48% (3 equivalents) was added dropwise to the solution kept in an ice bath under vigorous magnetic stirring. After 3 h, the bromide salt was precipitated by addition of dichloromethane. The powder was again dissolved in THF and precipitated with DCM, repeating the washing cycle three times. The resulting white powder of (NMA)Br was collected by filtration and dried under vacuum at 60 °C in a rotary evaporator.

### Synthesis of Europium Complex Eu(tta)_4_P(Ph)_4_

Eu(tta)_4_P(Ph)_4_ was synthetized following a previously reported procedure.^17^ Htta (20 mmol) was deprotonated with 20 mL of a 1 M NaOH water solution. P(Ph)_4_Cl (12 mmol) was dissolved in ethanol and added to the Htta solution. EuCl_3_·6H_2_O (5 mmol) was dissolved in ethanol and added dropwise to the ligand’s solution, causing the immediate precipitation of the complex. The resulting powder was filtered and washed three times with water and finally recrystallized from DCM (two times). The resulting light orange crystals were then recovered by filtration and dried under vacuum.

### Perovskite Synthesis

NMA_2_PbBr_4_ was synthetized by mixing (NMA)Br and PbBr_2_ in 2:1 M ratio in DMF to achieve the desired concentration (thin films for optical and XRD characterizations were obtained from 0.25 M solutions). For the doped perovskites NMA_2_PbBr_4_:Eu and NMA_2_PbBr_4_:Eu(L), EuCl_3_ or Eu(tta)_4_P(Ph)_4_ was, respectively, added to the precursor solution, both in the amount of 10% mol with respect to the lead content unless otherwise stated. All solutions were heated for 1 h at 100 °C and then spin-coated on fused silica substrates at 5000 rpm for 30 s. The films were finally annealed on a hotplate at 100 °C for 15 min. The entire procedure was performed in a glovebox under N_2_ atmosphere.

### Structural and Morphological Characterization

XRD experiments were done with a BRUKER D8 ADVANCE with Bragg–Brentano geometry, Cu Kα radiation (λ = 1.54056 Å), step increment of 0.02°, and 1 s of acquisition time. Morphological analyses and elemental mapping were carried out using an image-C_s_-corrected JEM-2200FS TEM (Schottky emitter), operated at 200 kV, in HAADF-STEM imaging mode. Elemental analysis and mapping were performed with a Bruker energy-dispersive X-ray spectrometer based on a XFlash-5060 SDD. Elemental quantification was done by the Cliff–Lorimer method using the L, K, L, K series for Pb, Br, Eu, and Cl, respectively. Integration of the same peaks was used for the elemental maps. Perovskite films were spin-coated on standard TEM silicon nitride windows on a silicon frame from Norcada (NT050X) (silicon thickness = 200 μm; nitride thickness 30 nm; window size 0.5 mm × 0.5 mm). The sample was mounted on an analytical TEM holder, with only Be components in the electron-irradiated region, in order to minimize the background in the EDS spectra.

### Spectroscopic Characterization

UV/VIS/NIR spectrophotometer λ 1050, PerkinElmer equipped with integrating sphere (module 150 mm InGaAs Int. Sphere) was used for absorption measurements. NanoLog (HORIBA Jobin Yvon) was used for steady-state PL measurements; absolute PLQY was measured using an integrating sphere (Quanta-ϕ) in combination with the NanoLog spectrofluorometer and using an excitation wavelength of 330 nm. Temperature- and pump fluence-dependent measurements were performed under nitrogen atmosphere using a Linkam Stage cooled with liquid nitrogen, exciting the sample with the third harmonic (355 nm) from a Nd:YAG Picolo-AOT laser (pulse length of approximately 1000 ps, 1 kHz repetition rate) focused on the sample with a 10 cm lens. PL was detected using a Maya1000 visible spectrometer. FTIR spectra were measured with a Bruker VERTEX 70 spectrometer in attenuated total reflectance mode with a Ge crystal. Measurements comprised 32 scans with 1 cm^–1^ resolution. For the analysis, perovskite films were deposited on silicon substrates by spin-coating precursor solutions with a concentration of 1 M in DMF. TRPL measurements on μs timescales were performed using an Andor iStar 320T ICCD-gated camera coupled to a Shamrock 303i spectrograph using a temporal step size of 100 μs and a spectral resolution of 0.2 nm. All measurements were performed in vacuum. As a pump, the third harmonic (355 nm) from a Nd:YAG Picolo-AOT laser was used, with a pulse length of 900 ps, repetition rate of 300 Hz, and pulse energy of 48 μJ/cm^2^. TRPL of the excitonic feature in the ps range was performed using a Hamamatsu streak camera and a Coherent Chameleon oscillator (pulse duration 30 fs, repetition rate 80 MHz) as a pump, using a pump wavelength λ = 380 nm, and a fluence *I*_pump_ = 790 mW/cm^2^, corresponding to a pulse energy of 9.87 nJ/cm^2^. The measurements on NMA_2_PbBr_4_ and NMA_2_PbBr_4_:Eu were performed using a measurement window 800 ps long, corresponding to a resolution of 8 ps as computed from the instrument response function (IRF). Measurements on NMA_2_PbBr_4_:Eu(L) were conducted using a 240 ps window, with a corresponding resolution of 2.8 ps. The excitonic emission lifetime was calculated by integrating the decay in the exciton spectral region (380–395 nm) and then fitting the normalized decay with a monoexponential gaussian decay *I*(*t*) = *I*_0_*e*^–(*t*–*t*_0_)/τ^ convoluted with the IRF; the fit parameters are reported in Table S1.

## Supporting Information Available

LED fabrication method, XRD data, optical characterization, STEM elemental maps, and electroluminescence measurements (PDF)

## Abbreviations

NMA: 1-naphtylmethylamine
tta: thenoyltrifluoroacetonate
Ph: phenyl
BA: butylammonium
PEA: phenethylammonium
HAADF: high-angle annular dark field
STEM: scanning transmission electron microscope
