# Supplementary material for: Layered Perovskite Doping with Eu3+ and β-diketonate Eu3+ Complex
Source: Chem Mater. 2021 Mar 21;33(7):2289–97. doi: 10.1021/acs.chemmater.0c04097 (PMC8043125; doi:10.1021/acs.chemmater.0c04097)
Supplement: Supplementary file 1 — cm0c04097_si_001.pdf [file cm0c04097_si_001.pdf]

Layered perovskite doping with Eu^{3+} and β-diketonate Eu^{3+} complex

Daniele Cortecchia,[1,*] Wojciech Mróz,[1] Giulia Folpini,[1] Tetiana Borzda,[1] Luca Leoncino,[2] Ada Lili Alvarado-Leaños,[1] Emily Mae Speller,[1] Annamaria Petrozza[1,*]

[1] *Centre for Nano Science and Technology (CNST@PoliMi), Istituto Italiano di Tecnologia, Milan, 20133, Italy*
[2] *Electron Microscopy Facility, Istituto Italiano di Tecnologia, Via Morego 30, 16163 Genova, Italy*
**Corresponding Authors: Annamaria.Petrozza@iit.it; Daniele.Cortecchia@iit.it*

1 Supplementary experimental method.

1.1 Materials.

Dimethylformamide DMF (anhydrous, Sigma Aldrich), chlorobenzene CB (anhydrous, Sigma Aldrich), poly(3,4-ethylenedioxythiophene) polystyrene sulfonate PEDOT:PSS (Heraeus Clevios P VP AI 4083), Poly[N,N'-bis(4-butylphenyl)-N,N'-bis(phenyl)-benzidine] PolyTPD (Ossila), 1,3,5-Tris(1-phenyl-1-H-benzimidazol-2-yl)benzene TPBi (Ossila), encapsulation resin LED401 (Master Bond Inc.).

1.2 Fabrication of light-emitting diodes (LEDs).

Glass substrates with indium tin oxide (ITO) were etched with Zn powder and 2M HCl. The etched substrates were cleaned ultrasonically in distilled water, acetone and isopropanol and subsequently O_2 plasma treated for 10 min. PEDOT:PSS (with thickness of 50 nm) was filtered with PVDF filters (0.22 µm) and spin coated at 4000 rpm, followed by thermal annealing at 110 °C for 10 min in air. After transferring the substrates in a N2 filled glovebox, PolyTPD (10 mg/ml in chlorobenzene) was spin-coated on PEDOT:PSS at 4000 rpm, followed by annealing at 100 °C for 10 min. PolyTPD had thickness of 25 nm. Before perovskite deposition, PolyTPD was exposed to O_2 plasma (5 s) to improve the film wettability. Subsequently, the perovskite NMA_2PbBr_4:Eu(L) was spin coated from hot DMF solution (100 °C) with concentration 0.04M using a rotation speed of 6000 rpm. The resulting film was annealed at 100 °C for 15 min achieving a thickness of approximately 30 nm. TPBi (35 nm) was thermally evaporated on the samples at a pressure $1*10^{-6}$ mbar. Finally, the cathode was deposited by transferring the samples to a metal evaporator, where 7 nm of barium and 100 nm of aluminium were evaporated through a shadow mask defining an active area of 9.3 mm^2. Completed devices were encapsulated inside N_2 filled glovebox with UV curable resin and a glass slide.

1.3 Device characterization.

Current–voltage (I–V) device characterization was performed with a Keithley 2614B source meter combined with a photodiode with known responsivity (OSI Optoelectronics, UV-100DQC). Measurements were realized by applying bias to the diode while the photodiode in contact with the device registered the emitted light. Electroluminescence was collected by an optical fiber and recorded by an Ocean Optics Maya 2000 Pro spectrometer. Efficiencies were calculated assuming a Lambertian distribution of emission.

2 Supporting tables and figures.

Table S1. Fit parameters and uncertainties for the lifetime of excitonic emission in NMA_2PbBr_4 samples with and without Eu doping.

	**NMA_2PbBr_4**	**NMA_2PbBr_4:Eu**	**NMA_2PbBr_4:Eu(L)**
**τ**	25.8 ± 0.85 ps	23.0± 0.9 ps	8.7 ± 0.3 ps
**I_0**	0.91 ± 0.02	0.91 ± 0.02	0.87 ± 0.02

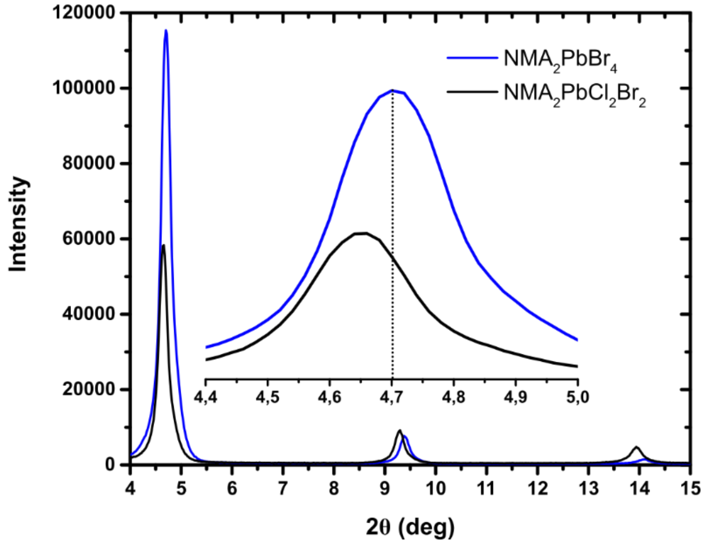

Figure S1. XRD pattern of NMA_2PbBr_4 (blue line) and $NMA_2PbCl_2Br_2$ (black line). Despite the smaller ionic radius of Cl^- (167 pm) compared to Br^- (182 pm), we find that chlorine addition causes a 0.052° shift of the XRD peaks to smaller angles corresponding to an increase in the interplanar distance from 18.75Å to 19.02Å. The addition of chlorine could induce differences in the coordination of the NMA^+ cations with consequent increase in the interplanar distance. Due to the strong preferential orientation of the perovskite which limits the number of diffraction peaks visible in the pattern, we cannot probe weather chlorine addition causes expansion or contraction along the other unit cell directions.

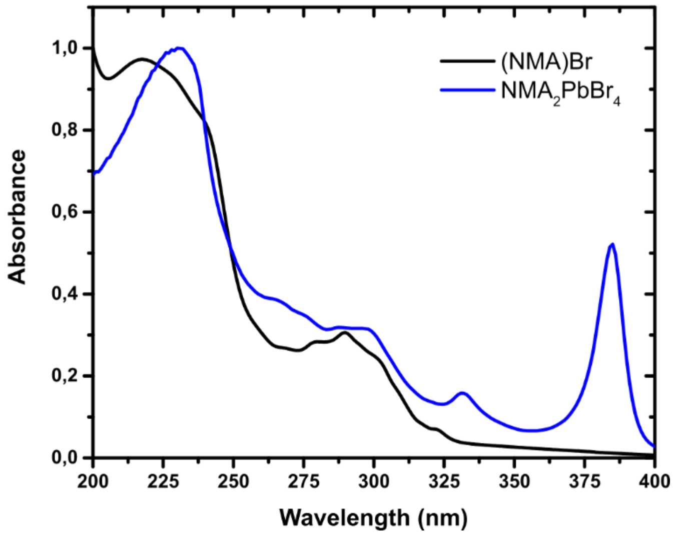

Figure S2. Absorption spectra of NMA_2PbBr_4 (blue line) and (NMA)Br (black line) thin films. The data show that the high-energy absorption of the perovskite is strongly related to the absorption of the organic cation.

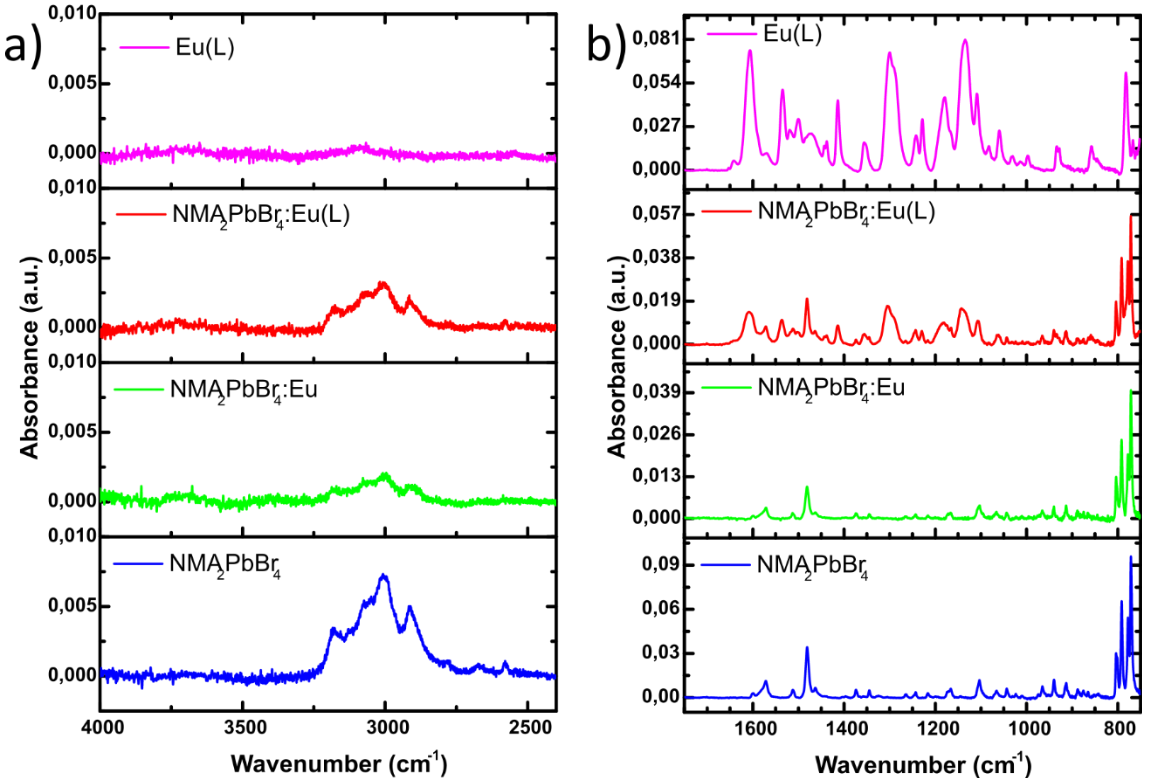

Figure S3. Fourier-transform infrared (FTIR) spectra of the doped and undoped NMA_2PbBr_4 perovskites and europium complex E(L). a) Envelope of the C-H aromatic and aliphatic stretching characteristic of NMA^+. b) Comparison of the IR absorption in the window 1750-750 cm^{-1}. While similar features are observed for NMA_2PbBr_4 and NMA_2PbBr_4:Eu, NMA_2PbBr_4:Eu(L) shows additional bands to those of the perovskite, which indicate the inclusion of the europium complex Eu(L) characterized by strong IR absorption bands.

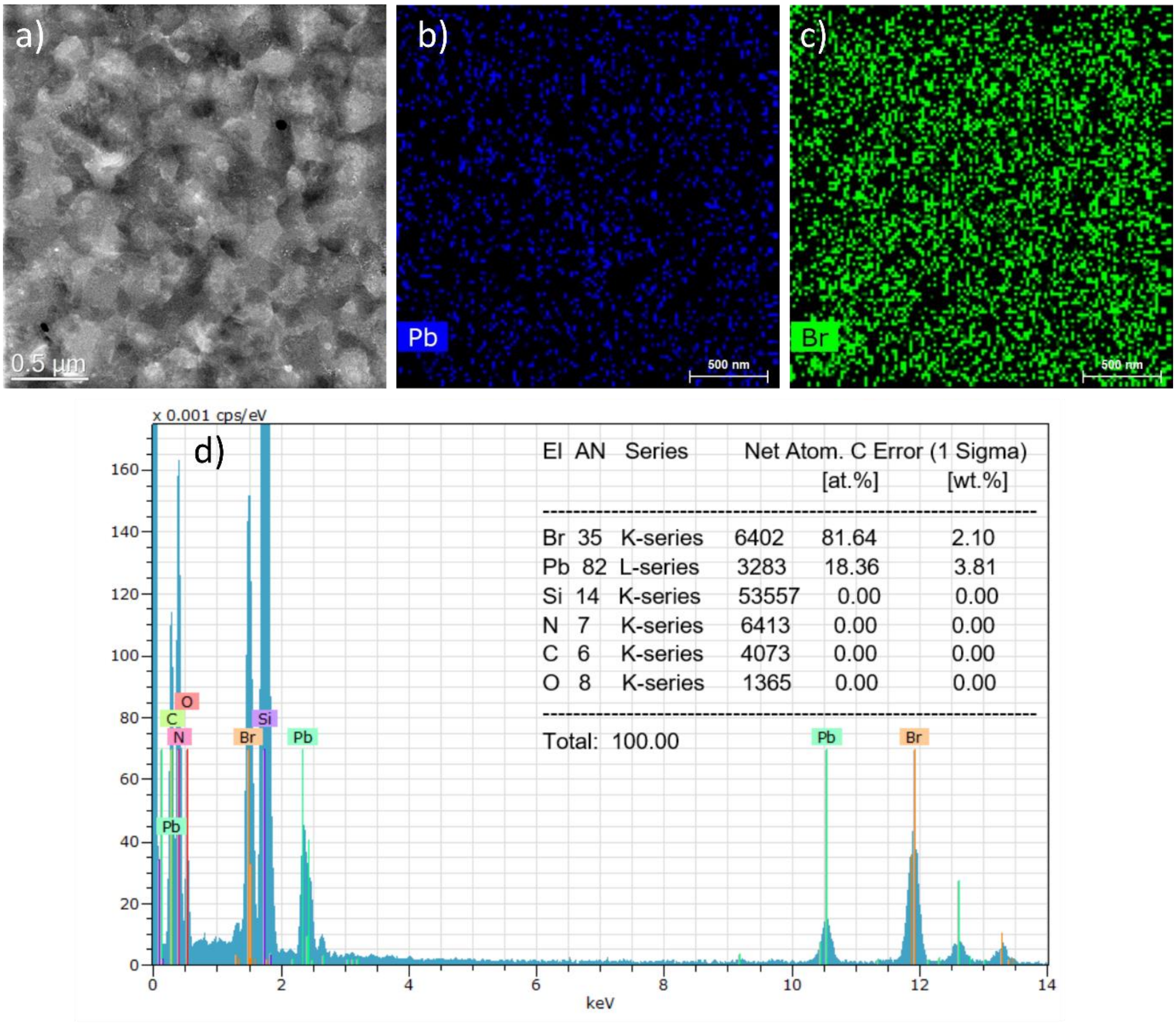

Figure S4. a) Representative HAADF-STEM image of the NMA_2PbBr_4 perovskite film and corresponding EDS elemental mapping of b) lead, c) bromine. d) EDS spectrum recorded from the whole area of the image. The Br[at%]/Pb[at%] ratio is 4.44 indicating that a slight excess of bromine is present respect to the nominal stoichiometry.

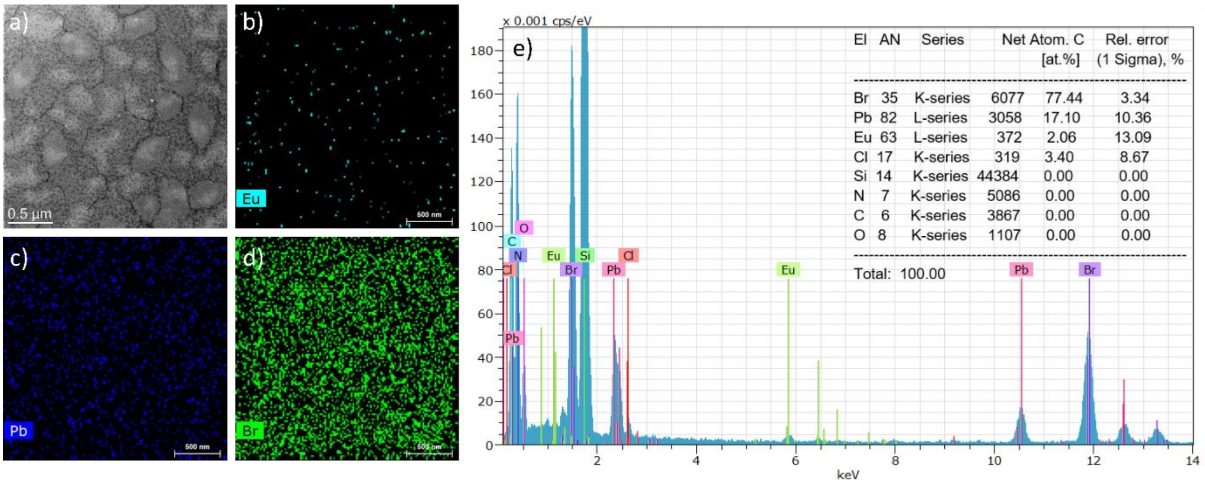

Figure S5. a) Representative HAADF-STEM image of the NMA_2PbBr_4:Eu perovskite film and corresponding EDS elemental mapping of b) europium, c) lead, d) bromine. e) EDS spectrum recorded from the whole area of the image. The Eu[at%]/Pb[at%] ratio is 12%, in slight excess than the expected 10%, and is homogenously distributed across the sample. Chlorine was also detected in the ratio Cl[at%]/Br[at%]=0.043, indicating a slight incorporation of chlorine deriving from $EuCl_3$.

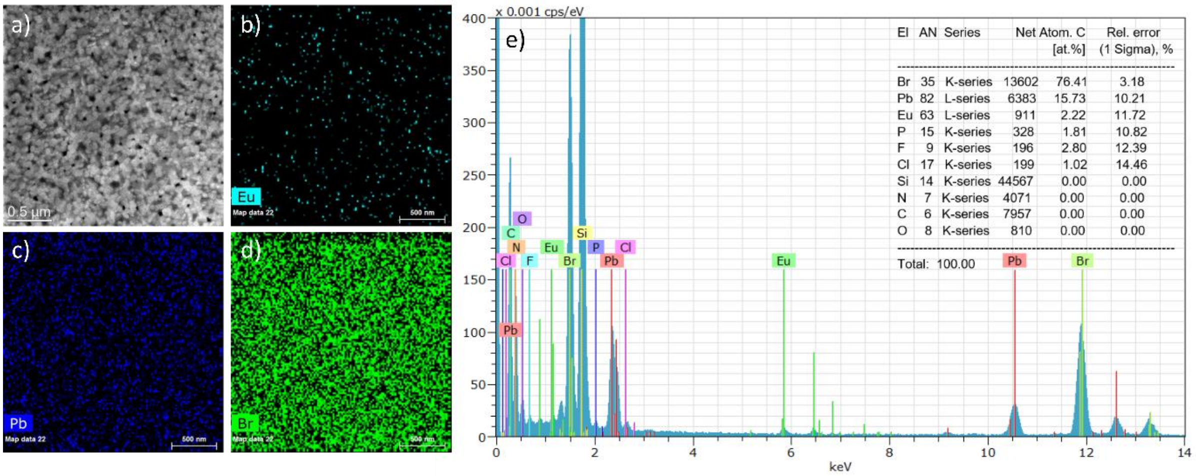

Figure S6. a) Representative HAADF-STEM image of the NMA_2PbBr_4:Eu(L) perovskite film and corresponding EDS elemental mapping of b) europium, c) lead, d) bromine. e) EDS spectrum recorded from the whole area of the image. The Eu[at%]/Pb[at%] ratio is 14.1%, in slight excess than the expected 10%, and is homogenously distributed across the sample. The EDS analysis also shows the presence of fluorine and phosphorous detected in the sample deriving from the europium complex and counter ion.

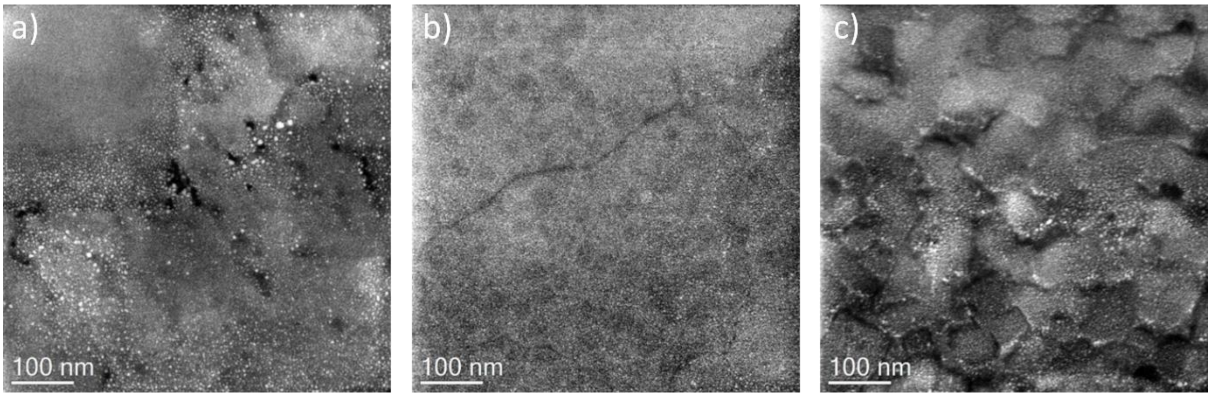

Figure S7. Higher magnification HAADF-STEM image of a) NMA_2PbBr_4, b) NMA_2PbBr_4:Eu, c) NMA_2PbBr_4:Eu(L) showing non uniformities at the sub μ-length scale with the presence of bright spots which can be attributed to Pb agglomerates/Pb nanoparticles.

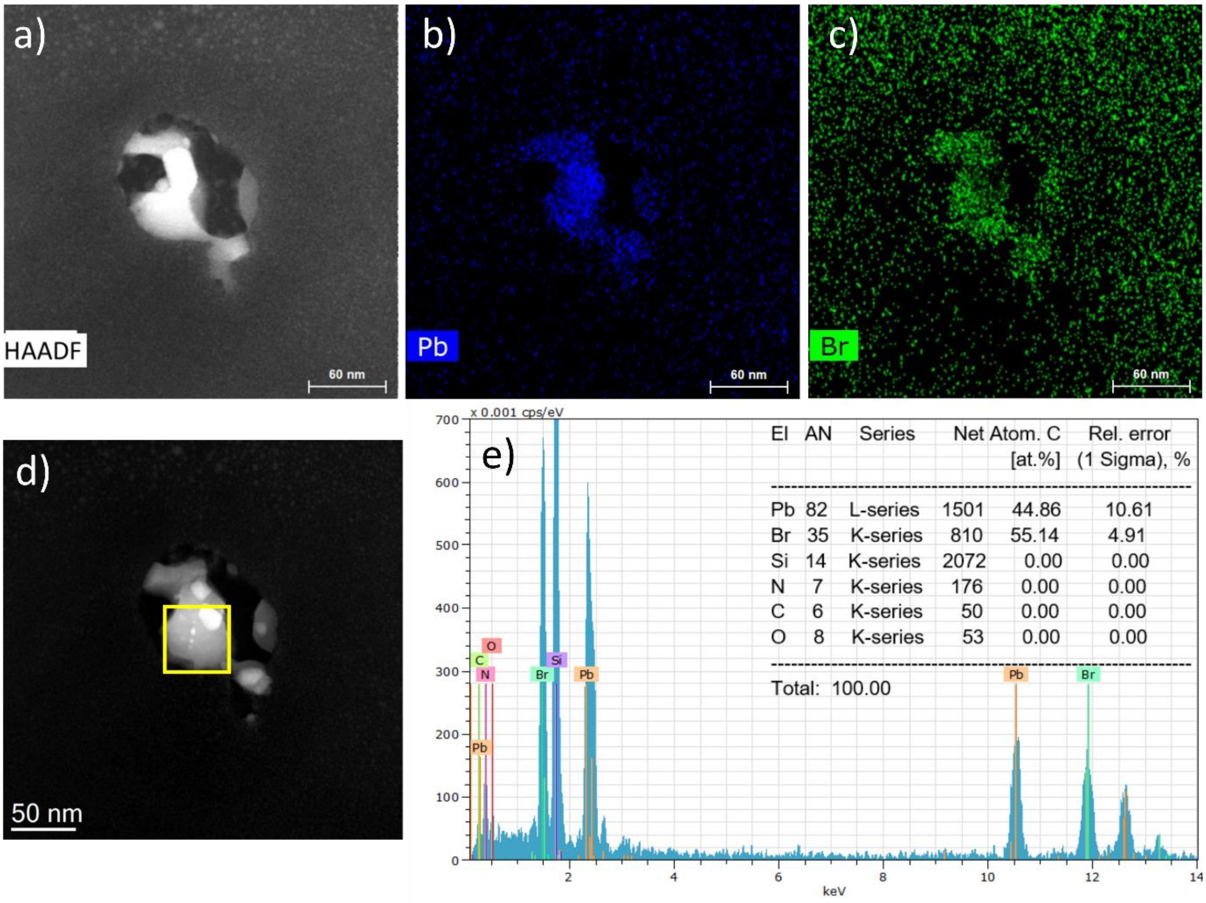

Figure S8. HAADF-STEM image of a Pb nanoparticle in NMA_2PbBr_4 and corresponding EDS elemental mapping of b) lead, c) bromine showing lead agglomeration matching with the nanoparticle position. d) HAADF-STEM image of Pb nanoparticles in NMA_2PbBr_4 and corresponding EDS spectrum (e) recorded from the highlighted central region, where the ratio Br[at%]/Pb[at%]=1.23 is indicative of a lead-reach area. Pb nanoparticles might derive from precipitation of metallic lead in the perovskite film.

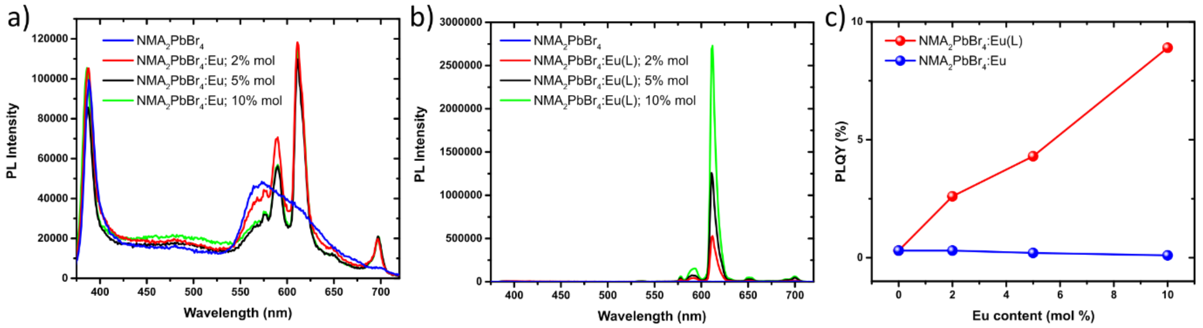

Figure S9. Photoluminescence spectra at different doping concentrations for a) NMA_2PbBr_4:Eu and b) NMA_2PbBr_4:Eu(L) and c) evolution of the photoluminescence quantum yield (PLQY) (λ_{exc} = 330 nm). Addition of Eu^{3+} causes a partial quenching of the NMA phosphorescence in favour of the lanthanide emission, but does not lead to an overall improvement of the luminescence yield. On the contrary, the addition of the europium complex results in a linear increase of the PLQY, reaching PLQY = 9% in the case of 10% molar content of Eu(L).

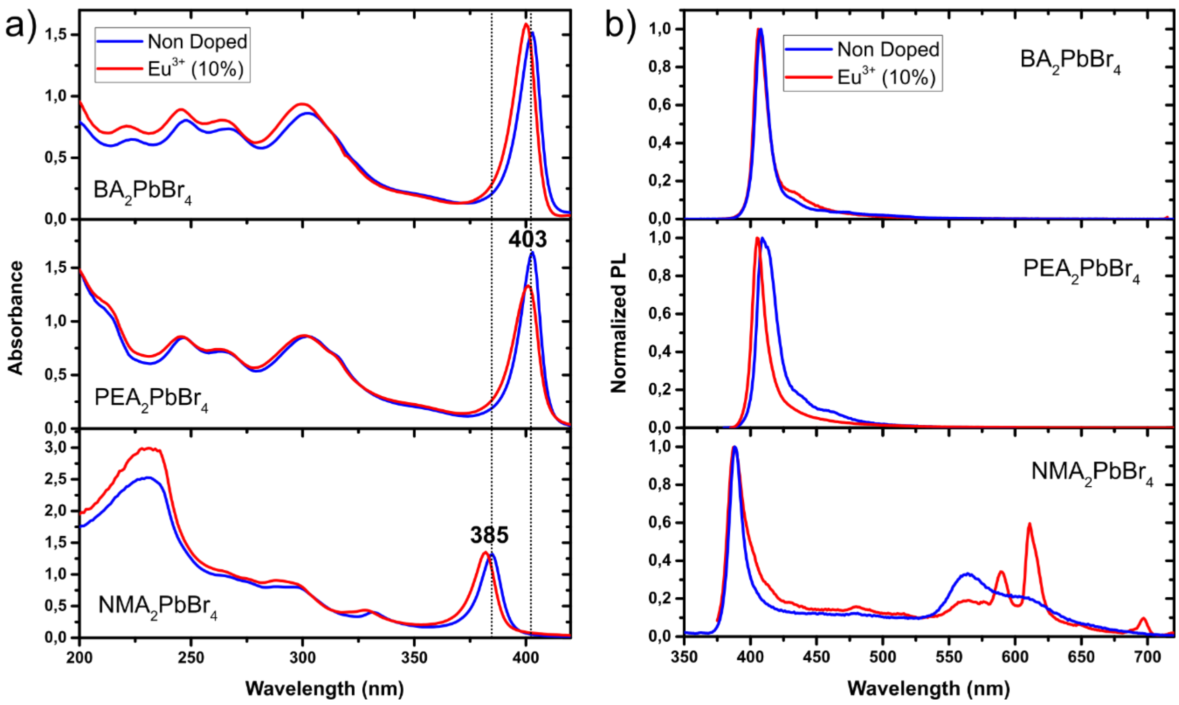

Figure S10. a) Absorption and b) photoluminescence spectra of BA_2PbBr_4 (upper panels) PEA_2PbBr_4 (middle panels) and NMA_2PbBr_4 (lower panels) where BA=butylammonium, PEA=phenetylammonium, NMA=1-napthylmethylammonium. Blue lines and red lines indicate the corresponding undoped and $EuCl_3$-doped materials. Eu^{3+} emission is observed only in $EuCl_3$-doped NMA_2PbBr_4, jointly with a partial quenching of the NMA phosphorescence at 563 nm.

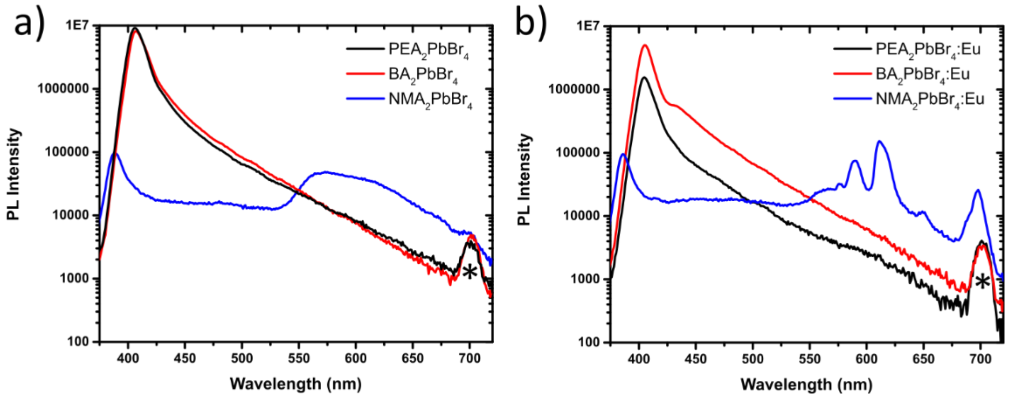

Figure S11. Photoluminescence spectra of a) PEA_2PbBr_4, BA_2PbBr_4 and NMA_2PbBr_4 and b) PEA_2PbBr_4:Eu, BA_2PbBr_4:Eu and NMA_2PbBr_4:Eu (λ_{ecx} = 350 nm). Data are shown in logarithmic scale. The peak marked with a star derives from the second order scattering of the pump, not perfectly cut by the long-pass filter. Europium emission is visible only in the NMA-based perovskite.

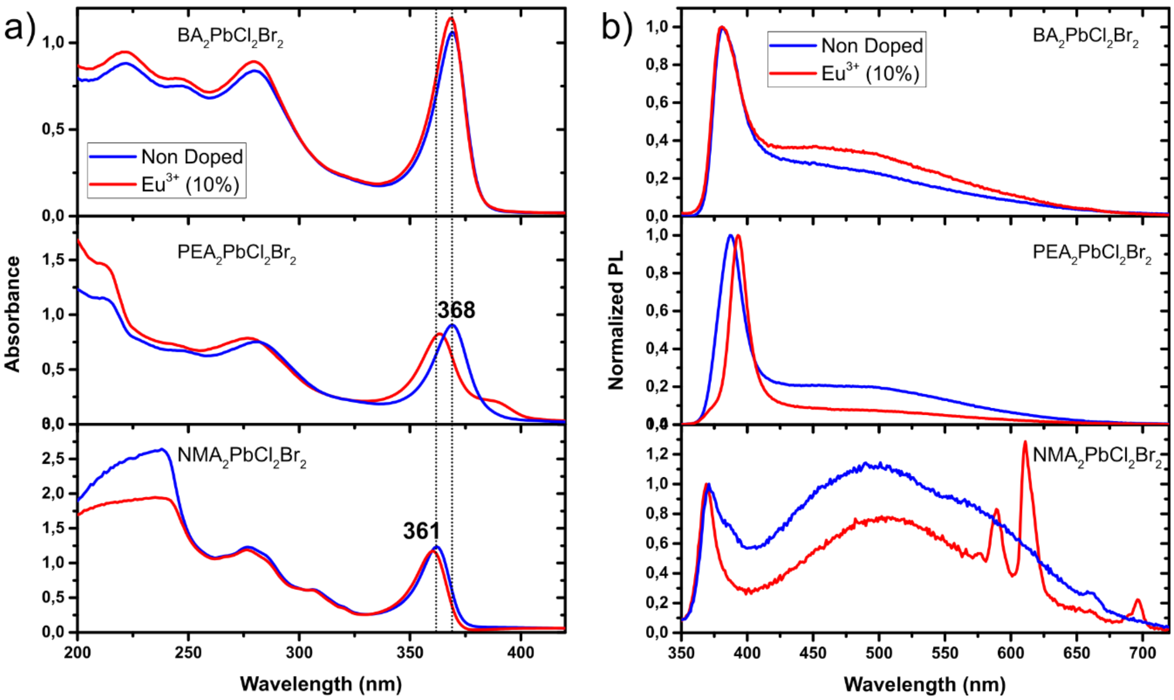

Figure S12. a) Absorption and b) photoluminescence spectra of $BA_2PbCl_2Br_2$ (upper panels) $PEA_2PbCl_2Br_2$ (middle panels) and $NMA_2PbCl_2Br_2$ (lower panels) where BA=butylammonium, PEA=phenetylammonium, NMA=1-napthylmethylammonium. With respect to the fully brominated perovskite, the perovskite band-gap is blue shifted, but largely Stokes shifted broadband PL concomitantly arise with the addition of chlorine likely related to self-trapped exciton emission. In the PEA-based perovskite, the presence of double excitonic peak is indicative of phase segregation occurring upon $EuCl_3$ addition, preventing the achievement of a clean shift of the band edge. Despite the increase in the band-gap with respect to the fully brominated compounds, the NMA-based perovskite is again the only one showing Eu^{3+} luminescence upon $EuCl_3$ doping.

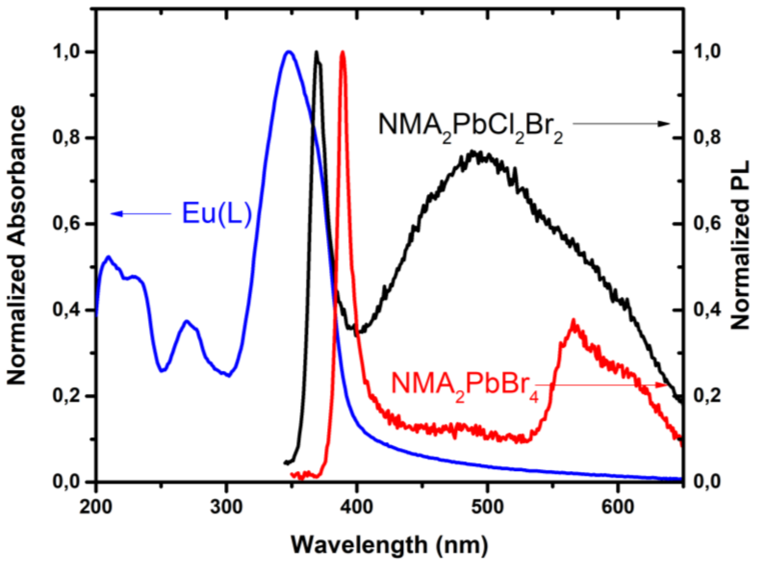

Figure S13. Absorption spectrum of the europium complex Eu(L) (blue line) compared to the PL spectra of NMA_2PbBr_4 (red line) and $NMA_2PbCl_2Br_2$ (black line). While NMA_2PbBr_4 PL overlaps only with the tail of Eu(L) absorption, better spectral overlap of the excitonic peak can be achieved exploiting the PL blue shift in $NMA_2PbCl_2Br_2$. However, excessive increase in chloride content leads to a great rise in the largely Stokes-shifted broadband PL deriving from exciton trapping phenomena, thus reducing the intensity of the excitonic emission. This, in turn, hampers a significant improvement of the spectral overlap through chlorine addition.

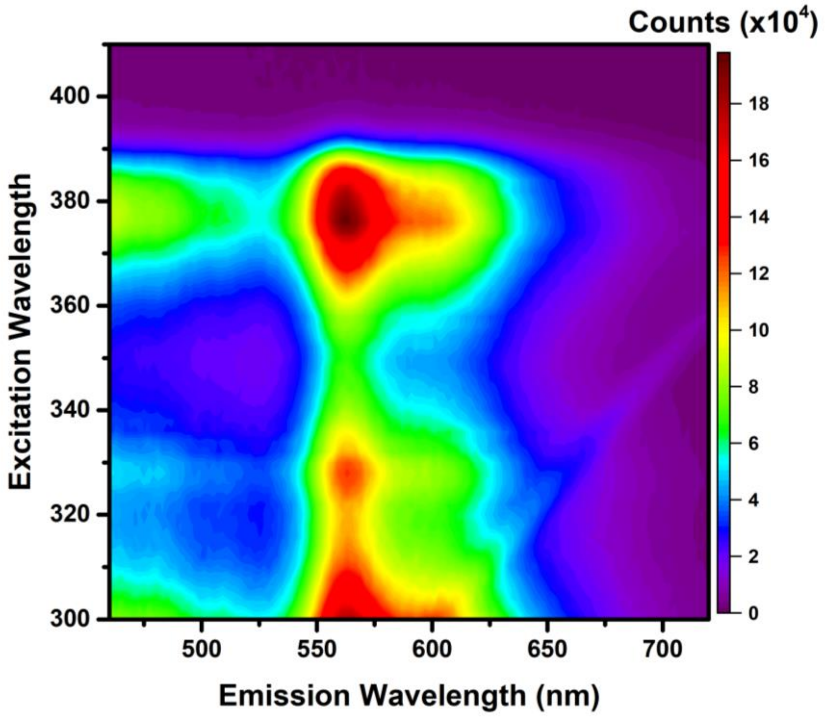

Figure S14. Photoexcitation spectrum of NMA_2PbBr_4, showing that the NMA phosphorescence perfectly traces the perovskite absorption thus proving that energy transfer is occurring between the $PbBr_4^{2-}$ inorganic layers and the NMA organic layers.

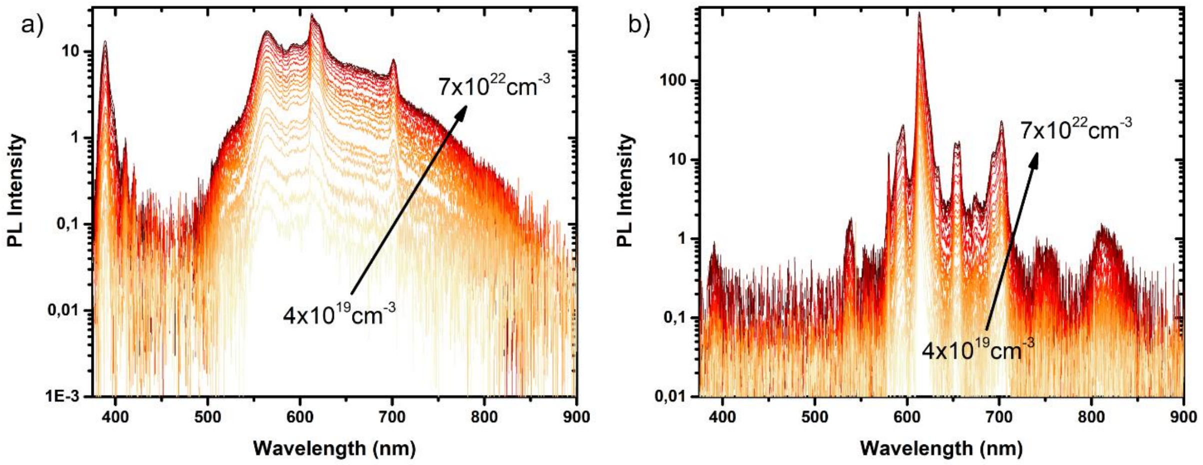

Figure S15. Excitation density dependent photoluminescence spectra (at 77 K) of a) NMA_2PbBr_4:Eu and b) NMA_2PbBr_4:Eu(L); the luminescence bands at 750 nm and 820 nm are characteristic of the weak europium *f-f* transitions $^5D_0 \rightarrow {}^7F_5$ and $^5D_0 \rightarrow {}^7F_6$. The band at 537 nm is likely to be related to emission from higher excited states such as $^5D_1 \rightarrow {}^7F_j$. The excitation wavelength is 355 nm, and the excitation density is scanned in the range 4x10^{19} cm^{-3} to 7x10^{22} cm^{-3}.

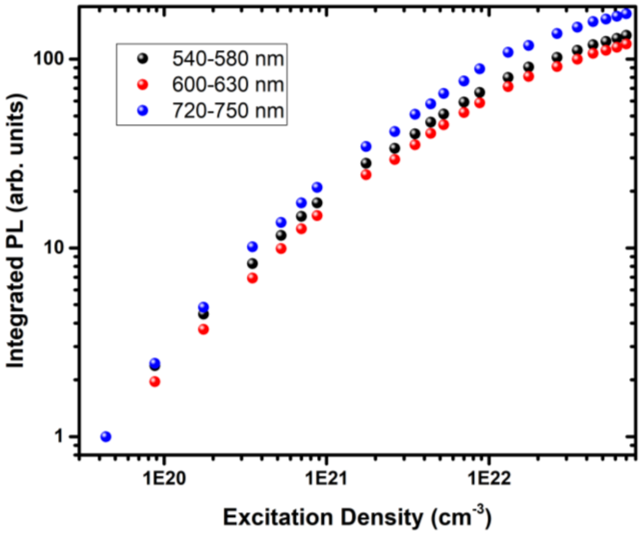

Figure S16. Integrated PL intensity, at 77 K, as function of excitation density for NMA_2PbBr_4:Eu. The different curves indicate different integration ranges: 540-580 nm (black), 600-630 nm (red), 720-750 nm (blue). Curves have been shifted on the y-axis to allow a better comparison.

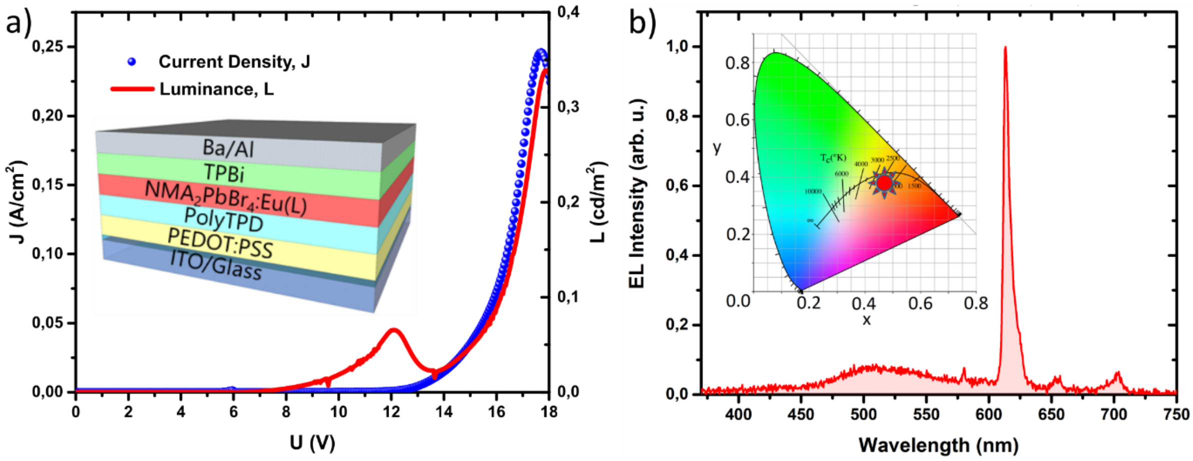

Figure S17. a) Device architecture (inset) and current density-voltage-luminance plot of the NMA_2PbBr_4:Eu(L) LED. The device architecture consists in the stack ITO/PEDOT:PSS/PolyTPD/NMA_2PbBr_4:Eu(L)/TPBi/Ba/Al, where PEDOT:PSS and PolyTPD are used as hole-injecting and hole-transporting materials, respectively, while TPBi is used as electron transporting layer. The slight mismatch between the luminance and the current density curve at the initial stage of operation and the high turn-on voltage can be ascribed to the limited transport properties of the wide band-gap 2D perovskite, where out-of-plane charge transport is hampered by the insulating organic layers. Small values of the luminance can be explained partially by the narrow electroluminescence (EL) spectrum. b) Electroluminescence spectrum of NMA_2PbBr_4:Eu(L) at 15V. The EL is dominated by the sharp ${}^5D_0 \rightarrow {}^7F_2$ hypersensitive transition at 611 nm, in excellent agreement with the PL spectrum NMA_2PbBr_4:Eu(L). This grants a line-like EL with exceptionally narrow full width at half maximum (FWHM = 6 nm) and Commission Internationale de l'Eclairage (CIE) coordinates x=0.47 and y=0.38 (inset). We also observed a weaker broad band between 450-600 nm, which is likely related to phosphorescence from the combined contributions of tta ligands and NMA templating cations.
